# Telitacicept in myasthenia gravis: a systematic review of clinical outcomes, steroid-sparing effects, and safety

**DOI:** 10.3389/fneur.2026.1916224

**Published:** 2026-08-25

**Authors:** Weitong Zhang, Yue Wang, Wenjun Qiao

**Affiliations:** 1 Liaoning University of Traditional Chinese Medicine, Shenyang, China; 2 Affiliated Hospital of Liaoning University of Traditional Chinese Medicine, Shenyang, China

**Keywords:** autoantibodies, BAFF, B-lymphocytes, myasthenia gravis, systematic review, telitacicept

## Abstract

**Introduction:**

Myasthenia gravis (MG) is an antibody-mediated autoimmune neuromuscular disorder for which long-term disease control remains challenging in some patients. Telitacicept, a dual inhibitor of B-cell activating factor (BAFF) and a proliferation-inducing ligand (APRIL), has been investigated as a potential therapeutic option for MG. This systematic review aimed to evaluate the clinical efficacy, steroid-sparing effects, and safety profile of telitacicept in patients with MG.

**Methods:**

A comprehensive search of PubMed, Scopus, Web of Science Core Collection, Embase, Cochrane Central Register of Controlled Trials (CENTRAL), China National Knowledge Infrastructure (CNKI), and Wanfang Data was conducted from database inception to January 19, 2026. Studies reporting clinical outcomes of telitacicept in MG were included. Two reviewers independently performed study selection, data extraction, and risk-of-bias assessment. Due to substantial heterogeneity in study designs, patient populations, treatment strategies, and outcome measures, a qualitative synthesis was performed.

**Results:**

Fifteen studies, including one randomized phase II trial, four retrospective cohort studies, one retrospective comparative study, four case series, and five case reports, were included. Available evidence generally suggested improvements in disease severity measures, particularly reductions in Quantitative Myasthenia Gravis (QMG) and Myasthenia Gravis Activities of Daily Living (MG-ADL) scores. In the randomized phase II trial of generalized MG, QMG decreased by approximately 7–10 points at 24 weeks, with response rates exceeding 90%. Several observational studies also reported reductions in glucocorticoid requirements. Adverse events were generally mild and transient, although the interpretation of long-term safety was limited by small sample sizes and short follow-up durations.

**Discussion:**

Current evidence suggests that telitacicept may provide potential clinical benefits and steroid-sparing effects in selected patients with MG. However, the certainty of evidence remains limited because most available studies were retrospective or uncontrolled, and further well-designed randomized controlled trials are required to establish its efficacy, long-term safety, and optimal clinical positioning.

## 1 Introduction

Myasthenia gravis (MG) is a neuromuscular autoimmune disease characterized by fluctuating muscle weakness and fatigue, mainly caused by autoantibody-mediated abnormalities of neuromuscular transmission (1). Pathogenic antibodies against postsynaptic membrane components, most commonly acetylcholine receptors (AChR) and muscle-specific kinase (MuSK), play a central role in the pathogenesis of the disease (2). Although traditional immunosuppressive and symptomatic treatments are effective for many patients, a considerable number of patients continue to suffer from refractory disease, treatment-related side effects, or long-term dependence on glucocorticoids (3). These problems highlight the urgent need for more targeted and sustainable treatment strategies (4, 5).

An in-depth understanding of the immune pathogenesis of MG has promoted the development of targeted biological therapies. Complement inhibitors and neonatal Fc receptor (FcRn) antagonists have demonstrated clinical benefit by blocking downstream effector mechanisms or accelerating IgG clearance (6–9). However, these approaches mainly target circulating antibodies or downstream immune pathways and do not directly address B-cell dysregulation that leads to persistent autoantibody production (10, 11). In addition, these therapies often require repeated administration and may impose substantial treatment costs, posing challenges for long-term disease treatment (12, 13).

Telitacicept is a recombinant fusion protein that simultaneously inhibits B-cell activating factor (BAFF) and a proliferation-inducing ligand (APRIL), which are key cytokines involved in B-cell survival, differentiation, and antibody production (14). By inhibiting BAFF and APRIL simultaneously, telitacicept may regulate early pathogenic B-cell responses and provide broader immune control compared with therapies targeting downstream signaling pathways (15, 16). Telitacicept has demonstrated clinical efficacy in a variety of antibody-mediated autoimmune diseases, including systemic lupus erythematosus and IgA nephropathy, which supports its potential therapeutic value in the treatment of MG (17–19).

In recent years, an increasing number of clinical studies have investigated the application of telitacicept in patients with MG, covering a variety of disease subtypes, including generalized, ocular, thymoma-associated, pediatric, and refractory MG. Early clinical trials, retrospective cohort studies, and case reports have suggested that telitacicept may improve disease severity, reduce steroid requirements, and have acceptable short-term safety. For highly refractory cases, there have also been reports of sequential or combination treatment strategies involving telitacicept (20–22). However, the existing data are heterogeneous in study design and mainly derived from small observational studies and case reports, making it difficult to draw definitive conclusions. Therefore, a systematic evaluation of the current evidence is needed to better characterize the efficacy, steroid-sparing effects, safety profile, and potential clinical role of telitacicept in MG.

## 2 Methods

### 2.1 Search strategy

This review was conducted in accordance with the PRISMA 2020 (Preferred Reporting Items for Systematic Reviews and Meta-Analyses) guidelines (23). A comprehensive literature search was conducted in PubMed, Scopus, Web of Science Core Collection, Embase, Cochrane Central Register of Controlled Trials (CENTRAL), China National Knowledge Infrastructure (CNKI), and Wanfang Data. The search covered all studies published from database inception to January 19, 2026, without language restrictions. The search strategy combined controlled vocabulary terms (e.g., MeSH and Emtree) and free-text keywords related to MG and telitacicept. The search strategy was adapted for each database. Detailed search strategies for each database are provided in Supplementary Table S5. Reference lists of included studies and relevant reviews were also manually screened to identify additional potentially relevant publications.

### 2.2 Eligibility criteria

Studies meeting the following criteria were included: (1) patients diagnosed with MG; (2) treatment with telitacicept as monotherapy, add-on therapy, or part of a sequential or combination treatment strategy; (3) reporting clinical outcomes such as disease severity [e.g., Quantitative Myasthenia Gravis (QMG) and Myasthenia Gravis Activities of Daily Living (MG-ADL)], quality of life, steroid-sparing effects, or safety outcomes; and (4) original clinical studies, including randomized controlled trials, cohort studies, case series, and case reports.

Studies were excluded if they were review articles, editorials, or comments without original data; basic or translational studies; studies investigating interventions other than telitacicept; or registered or ongoing clinical trials without published peer-reviewed results.

### 2.3 Study selection

All retrieved references were imported into reference management software, and duplicates were removed. Two reviewers independently screened the titles and abstracts and then evaluated the full texts of potentially relevant studies according to the predefined criteria. Disagreements between the reviewers were resolved through discussion, and a third reviewer was consulted if necessary. Potentially related publications were retained during the selection process for further assessment of participant overlap rather than being excluded *a priori*.

### 2.4 Data extraction and qualitative evidence synthesis

Data extraction was independently performed by two reviewers using a standardized data collection form. The extracted data included study characteristics, patient characteristics, treatment details, efficacy outcomes, safety outcomes, and information relevant to the assessment of potential participant overlap. The extracted information is summarized in Tables 1, 2 and Supplementary Tables S1–S4, with potential participant overlap assessments provided in Supplementary Table S6.

**Table 1:** Characteristics of studies included in the systematic review.

First author (year)	Country	Study design	Myasthenia gravis subtype	Sample size (n)	Telitacicept regimen	Follow-up duration
Yin et al. (2024) (27)	China	Multicenter, randomized, open-label phase 2 clinical trial	Generalized MG	29	Telitacicept 160/240 mg + SOC	24 wks
Lin et al. (2026) (31)	China	Single-center retrospective case series	Ocular MG (refractory)	7	Telitacicept 80–160 mg (add-on)	Med 7 mo (5–8)
Lu et al. (2025) (28)	China	Single-center retrospective cohort study (PSM)	Mixed MG	28 (14 vs. 14)	Telitacicept 160 mg + SOC	24 wks
Zhang et al. (2025) (21)	China	Single-center retrospective case series	Generalized MG	7	Efgartigimod → Telitacicept 240 mg + SOC	28 wks
Lin et al. (2024) (22)	China	Single-center retrospective cohort study	Generalized MG (refractory)	16	Telitacicept 160 mg (add-on)	3–6 mo
Mao et al. (2025) (32)	China	Single-center retrospective case series	Ocular MG (pediatric)	4	Telitacicept 80 mg (add-on)	4–12 mo
Yang et al. (2025) (34)	China	Case reports	MG (with CTD)	3	Telitacicept 160 mg (add-on)	Up to 12 mo
Song et al. (2025) (33)	China	Case series	Generalized MG (refractory)	4	Telitacicept 160 mg monotherapy	12 wks
Zhang et al. (2024) (37)	China	Case reports	Generalized MG (refractory)	2	Telitacicept 160 mg (add-on)	16 wks
Qiu and Ying (2025) (35)	China	Case report	Generalized MG (with RA)	1	Telitacicept 160 mg (add-on)	~2 mo
Wang et al. (2024) (36)	China	Case report	Generalized MG (MuSK-positive, refractory)	1	Telitacicept 160 mg + RTX	Up to 15 mo
Zhang et al. (2024) (38)	China	Case report	Generalized MG (refractory)	1	Telitacicept 160 mg + Efgartigimod	~30 wks
Dou (2025) (29)	China	Single-center retrospective cohort study	Generalized and ocular MG	39	Telitacicept 160 mg + SOC	6 mo
Zhu et al. (2026) (30)	China	Single-center retrospective cohort study	Generalized MG	22	Telitacicept 160 mg (add-on)	6–12 mo
Fang et al. (2025) (20) – telitacicept	China	Single-center retrospective cohort study	Generalized and ocular MG	10	Telitacicept 160/240 mg monotherapy	5 mo
Fang et al. (2025) (20) – sequential therapy	China	Single-center retrospective cohort study	Generalized MG	6	Efgartigimod → Telitacicept 240 mg	21 wks

MG, myasthenia gravis; SOC, standard of care; PSM, propensity score matching; CTD, connective tissue disease; RA, rheumatoid arthritis; RTX, rituximab; wks, weeks; mo, months; Med, median. + SOC: telitacicept added to a prespecified standard-of-care regimen. Add-on: telitacicept administered in addition to patients’ existing, non-standardized background therapies. Monotherapy: telitacicept used without concurrent immunosuppressants. →: sequential therapy.

**Table 2:** Summary of efficacy outcomes reported across included studies.

Study (author, year)	Study design	Outcome measure(s)	Direction of change	Key efficacy findings	Follow-up time point(s)
Yin et al. (2024) (27)	Multicenter, randomized, open-label phase 2 clinical trial	QMG score; MG clinical absolute score; QMG responder rate; IgG/IgA/IgM; CD19^+^ B cells	Decreased (QMG, MG clinical absolute score, IgG/IgA/IgM); increased and decreased over time (CD19^+^ B cells)	Mean QMG reduction from baseline to week 24 was −7.7 (160 mg) and −9.6 (240 mg); at week 12: −5.8 and −9.5. QMG responder rate at week 24 reached 92.9 and 100.0%, respectively. MG clinical absolute score and serum IgG, IgA, and IgM levels decreased over 24 weeks.	Wks 4, 8, 12, 16, 20, 24
Lin et al. (2026) (31)	Single-center retrospective case series	MG-ADL; QMG; MGII-PRO; MGFA-PIS (including MSE)	Decreased (MG-ADL, QMG, MGII-PRO); increased (MSE achievement)	A ≥ 2-point MG-ADL improvement was achieved in 6/7 patients by the third follow-up. At 3 months, 6/7 patients showed significant improvement in MG-ADL, MGII-PRO, and QMG scores. Among responders, 4/6 achieved MSE, and these improvements were sustained during follow-up.	3, 6 mo
Lu et al. (2025) (28)	Single-center retrospective cohort study (PSM)	MG-ADL; QMG; MMS; corticosteroid dose; CD19^+^ B cells	Decreased (MG-ADL, QMG, corticosteroid dose, CD19^+^ B cells); increased (MMS achievement rate)	MG-ADL and QMG scores decreased significantly at weeks 4, 12, and 24. MMS achievement increased over time. Corticosteroid dose was reduced, and CD19^+^ B cell counts decreased during follow-up.	4, 12, 24 wks
Zhang et al. (2025) (21)	Single-center retrospective case series	QMG; MG-ADL; MMS; prednisone dose; IgG/IgA/IgM; CD19^+^ B cells; BAFF; APRIL	Decreased (QMG, MG-ADL, prednisone dose, IgA/IgM); increased (MMS, BAFF); stable (IgG, APRIL); fluctuated (CD19^+^ B cells)	QMG and MG-ADL decreased significantly by weeks 13 and 28. Six of seven patients (86%) achieved MMS, with a median time of 9 weeks. Mean daily prednisone dose decreased markedly by week 28. IgA and IgM decreased, whereas IgG and APRIL remained stable and BAFF increased during follow-up.	13, 28 wks
Lin et al. (2024) (22)	Single-center retrospective cohort study	MGFA-PIS; MGSTI; QMG; MG-ADL; daily prednisone dose	Decreased (MGSTI, QMG, MG-ADL, daily prednisone dose); increased (MMS/PR achievement)	At 3 months, 75.0% (12/16) showed clinical improvement, including one PR and two MMS. At 6 months, 90.1% (10/11) maintained improvement. MGSTI, QMG, and MG-ADL decreased significantly, and prednisone dose was reduced during follow-up.	3, 6 mo
Mao et al. (2025) (32)	Single-center retrospective case series	Clinical symptoms; QMG; MG-ADL; CD19^+^ B cells; IgG/IgA/IgM; medication adjustment	Decreased (QMG, MG-ADL, CD19^+^ B cells, IgG/IgA/IgM)	All four patients showed improvement in ocular symptoms. QMG and MG-ADL scores decreased at the last follow-up. All patients discontinued glucocorticoids and other immunosuppressants. CD19^+^ B cell percentages and immunoglobulin levels showed a decreasing trend.	Last f/u (4–12 mo)
Yang et al. (2025) (34)	Case reports	QMG; MG-ADL; MSE; prednisone dose	Decreased (QMG, MG-ADL, prednisone dose); increased (MSE)	Case 1: QMG 13 → 2; MG-ADL 10 → 1. Case 2: QMG 21 → 1; MG-ADL 19 → 0. Case 3: QMG 23 → 3; MG-ADL 15 → 1. All patients achieved MSE within 6–8 weeks.	Last f/u (6–12 mo)
Song et al. (2025) (33)	Case series	MGFA-QMG; MG-QOL-15; MG-ADL; CD19^+^ B cells; AChR-Ab; IgG/IgA/IgM	Decreased (QMG, MG-QOL-15, MG-ADL, CD19^+^ B cells, AChR-Ab, IgG/IgA/IgM)	All patients showed marked improvement during the 8-week treatment. MGFA-QMG, MG-QOL-15, and MG-ADL decreased. CD19^+^ B cell counts, AChR-Ab, and immunoglobulin levels declined during treatment.	Wks 2, 4–5, 8, 12
Zhang et al. (2024) (37)	Case reports	QMG; MG-ADL; MGFA; memory B cells; plasmablasts; plasma cells; BLyS; APRIL; IgG; BCMA; AChR-Ab	Decreased (QMG, MG-ADL, IgG, AChR-Ab, B-cell subsets)	Both patients showed rapid clinical improvement within 2 weeks. By week 8, QMG and MG-ADL scores decreased markedly. Immunological markers showed an overall downward trend during treatment.	Wks 8, 12–16
Qiu and Ying (2025) (35)	Case report	MG symptoms; ocular deviation; CRP; ESR; DAS28-ESR; joint ultrasound	Decreased (CRP, ESR, DAS28-ESR); improved symptoms	Clinical improvement was observed after 2 months. CRP, ESR, and DAS28-ESR decreased, and joint ultrasound showed improvement in synovitis.	~1 mo
Wang et al. (2024) (36)	Case report	QMG; MG-ADL; MuSK-Ab titer; CD19^+^ lymphocyte; B-cell percentage; IgG/IgA/IgM	Decreased (QMG, MG-ADL, MuSK-Ab, CD19^+^ B cells, IgG/IgA/IgM)	QMG and MG-ADL decreased within 2 weeks. After 8 injections, further improvement was observed. Following relapse, combination therapy maintained improvement, and MuSK-Ab titer decreased.	Wk 8 (initial); 3 mo post-combo
Zhang et al. (2024) (38)	Case report	QMG; MG-ADL; MGC; prednisone dose; pyridostigmine dose; serum B-cell count; lymphocyte count; Ig levels	Decreased (QMG, MG-ADL, MGC, prednisone dose, pyridostigmine dose, immunological markers)	QMG and MG-ADL decreased by 8 and 12 points after 4 weeks. Continued improvement was observed after combination therapy. Prednisone and pyridostigmine doses were reduced, and no myasthenic crisis occurred.	Wks 4, 16, 28–30
Dou (2025) (29)	Single-center retrospective cohort study	QMG; MG-ADL; MGC; prednisone dose; CD19^+^ B cells; IgG/IgA/IgM	Decreased (QMG, MG-ADL, MGC, prednisone dose, CD19^+^ B cells, IgG/IgA/IgM)	QMG, MG-ADL, and MGC decreased significantly at 1, 3, and 6 months. Prednisone dose was reduced, and CD19^+^ B cells and immunoglobulin levels decreased during follow-up.	1, 3, 6 mo
Zhu et al. (2026) (30)	Single-center retrospective cohort study	QMG; MG-ADL; MG-QOL-15; MSE; prednisone dose; AChR-Ab; CD19^+^ B cells; IgG/IgA/IgM	Decreased (QMG, MG-ADL, MG-QOL-15, prednisone dose, AChR-Ab, CD19^+^ B cells, IgG/IgA/IgM); increased (MSE)	QMG decreased from 15.68 ± 5.42 to 5.59 ± 3.53 at 3 months. MG-ADL and MG-QOL-15 also decreased significantly. 20/22 patients achieved MSE (median 4 months), and prednisone was reduced to ≤5 mg/day.	Wks 4, 12, 24 & last f/u
Fang et al. (2025) (20)	Single-center retrospective cohort study	QMG; MG-ADL; MSE; prednisone dose	Decreased (QMG, MG-ADL, prednisone dose); increased (MSE)	In the monotherapy cohort, 8/10 patients achieved MSE within 4 months, with decreased QMG and MG-ADL scores. In the sequential therapy cohort, MG-ADL improved after induction therapy, and further reduction in prednisone dose was observed.	2, 4 mo (telitacicept); Wks 4–21 (sequential therapy)

MG, myasthenia gravis; PSM, propensity score matching; QMG, Quantitative Myasthenia Gravis; MG-ADL, Myasthenia Gravis Activities of Daily Living; MGFA-PIS, Myasthenia Gravis Foundation of America Post-Intervention Status; MGII-PRO, Myasthenia Gravis Impairment Index–Patient-Reported Outcome; MGSTI, Myasthenia Gravis Status and Treatment Intensity; MGC, Myasthenia Gravis Composite; MG-QOL-15, 15-item Myasthenia Gravis Quality of Life; MMS, minimal manifestation status; MSE, minimal symptom expression; PR, pharmacological remission; Ig, immunoglobulin; IgG, immunoglobulin G; IgA, immunoglobulin A; IgM, immunoglobulin M; AChR-Ab, acetylcholine receptor antibody; MuSK-Ab, muscle-specific kinase antibody; BAFF, B-cell activating factor; APRIL, a proliferation-inducing ligand; BLyS, B lymphocyte stimulator; BCMA, B-cell maturation antigen; CRP, C-reactive protein; ESR, erythrocyte sedimentation rate; DAS28-ESR, Disease Activity Score in 28 Joints–Erythrocyte Sedimentation Rate; f/u, follow-up; wks, weeks; mo, months. †In the “Direction of change” column, direction relative to baseline is indicated. For studies with two arms, values are presented as telitacicept group vs. control group where applicable.

Given the substantial heterogeneity in study designs, patient populations, treatment strategies, and reported outcome measures, quantitative pooling was considered inappropriate. Therefore, a qualitative evidence synthesis was conducted following the Synthesis Without Meta-analysis (SWiM) reporting guideline (24). The synthesis was structured according to clinically relevant domains, including efficacy outcomes, steroid-sparing effects, safety outcomes, and treatment strategies (monotherapy, add-on therapy, and sequential or combination approaches), with sequential or combination treatment strategies analyzed separately where appropriate.

### 2.5 Risk of bias assessment

The methodological quality and risk of bias of included studies were assessed according to study design. The randomized controlled trial was evaluated using the Cochrane Risk of Bias tool (RoB 2) (25). Retrospective cohort and comparative studies were evaluated using the Newcastle–Ottawa Scale (NOS). Case reports and case series were evaluated using the Joanna Briggs Institute (JBI) critical appraisal tools (26). Given the substantial heterogeneity in study designs and the predominance of observational evidence, the overall strength and certainty of the available evidence were considered qualitatively based on study design, risk of bias, consistency of findings, and limitations of the available evidence.

### 2.6 Assessment of potential participant overlap

Potential participant overlap among included studies was additionally assessed by comparing study centers, recruitment periods, investigator groups, patient populations, and treatment characteristics. Because individual participant-level information was unavailable, potential participant overlap could neither be confirmed nor completely excluded; therefore, studies were categorized according to the likelihood of potential overlap. The detailed assessment is provided in Supplementary Table S6.

## 3 Results

### 3.1 Study selection

**Figure 1:**
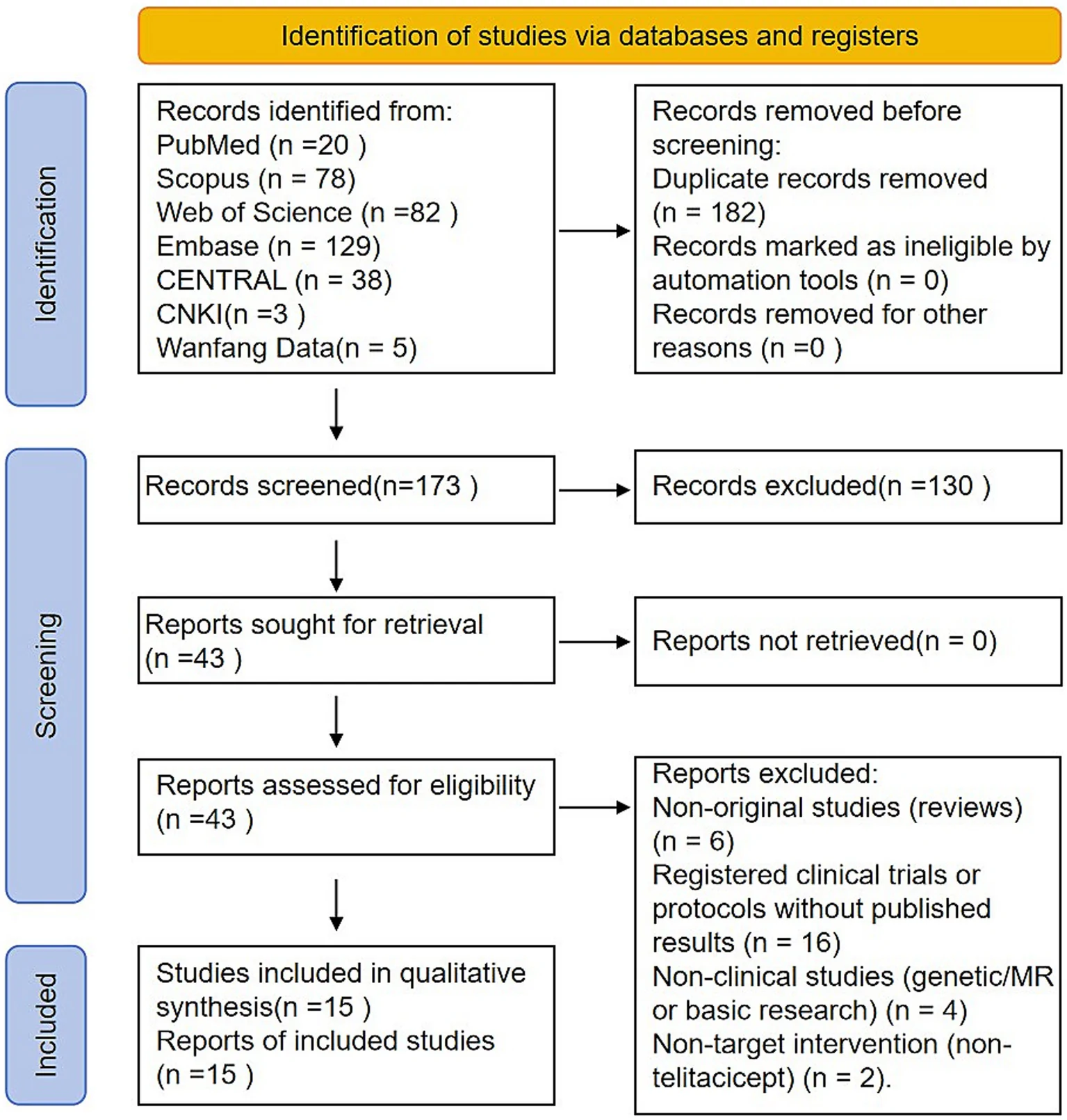
PRISMA 2020 flow diagram of study selection.

A total of 355 records were identified through database searching. After removing 182 duplicate records, 173 records remained for title and abstract screening, of which 130 were excluded as irrelevant. The remaining 43 articles underwent full-text review, and 28 were excluded for predefined reasons, including review articles without original clinical data, registered or ongoing studies without published peer-reviewed results, non-clinical studies, and studies involving non-target interventions. Finally, 15 studies met the inclusion criteria and were included in the qualitative synthesis (Figure 1). Potential partial overlap could not be completely excluded for two publications from the Wenzhou Medical University group reporting sequential efgartigimod–telitacicept therapy; therefore, these studies were interpreted cautiously during qualitative synthesis.

### 3.2 Characteristics of included studies

The characteristics of the included studies are summarized in Table 1 and Supplementary Table S1. The 15 included studies consisted of one multicenter randomized open-label phase 2 clinical trial (27), four retrospective cohort studies (22, 28–30), one retrospective comparative study (20), four case series (21, 31–33), and five case reports (34–38). One study (20) involved two independent cohorts: one cohort received telitacicept monotherapy (*n* = 10), while the other cohort received sequential therapy (*n* = 6).

All studies were conducted in China and published between 2023 and 2026. The sample sizes ranged from single-patient case reports to cohort studies involving up to 39 patients treated with telitacicept. The study populations were clinically heterogeneous and included generalized MG, refractory generalized MG, refractory ocular MG, thymoma-associated MG, pediatric ocular MG, and connective tissue disease-associated MG. The dosage of telitacicept ranged from 80 to 240 mg weekly. Most studies used telitacicept as add-on therapy to conventional treatment regimens, while two studies used telitacicept as monotherapy (20, 33) (monotherapy cohort). Sequential or combination treatment strategies involving efgartigimod or rituximab were reported in four studies. Follow-up duration ranged from approximately 2 to 15 months depending on the study (Table 1).

Baseline patient characteristics are summarized in Supplementary Table S2. There was substantial heterogeneity in age, disease duration, antibody status, and disease severity. The mean or median age ranged from 10.4 years in pediatric ocular MG to 70 years. Disease duration ranged from several months to more than 10 years. Most patients had generalized MG, while some patients had ocular or refractory MG. Most patients were positive for acetylcholine receptor antibodies (AChR-Ab), while a small number of patients were positive for MuSK antibodies or seronegative. Among studies reporting MGFA classification, disease severity ranged from class I to class V. Among studies reporting QMG scores, baseline values ranged from approximately 0–4 in pediatric ocular MG to approximately 18–20 in refractory disease. Most patients received combination therapy including pyridostigmine, corticosteroids, and immunosuppressants, reflecting routine clinical practice.

### 3.3 Clinical efficacy

Available evidence from the included studies generally suggested improvements in disease severity measures, particularly reductions in QMG and MG-ADL scores. Where available, clinically meaningful response definitions reported in the original studies, such as QMG response (defined as a ≥ 3-point reduction), minimal manifestation status (MMS), minimal symptom expression (MSE), and minimal remission (MR, according to the criteria used in the original study), were considered in the interpretation of treatment effects. However, standardized responder thresholds or minimal clinically important differences (MCIDs) were not consistently reported across studies, limiting direct comparison of clinical significance among different outcome measures. The certainty of these findings varied according to study design, with the randomized phase II trial and retrospective cohort/comparative studies providing relatively stronger support for evaluating clinical effects, whereas uncontrolled studies mainly contributed supportive evidence from diverse clinical settings. In a multicenter, randomized, phase II study (27), the mean QMG score decreased by 7.7 points in the 160 mg group and 9.6 points in the 240 mg group from baseline to week 24. By week 24, the QMG response rates (defined as a reduction of ≥3 points) reached 92.9 and 100% in the two groups, respectively. During the 24-week treatment period, reductions in MG absolute clinical scores were observed, accompanied by decreases in serum immunoglobulin concentrations (IgG, IgA, and IgM) (27). Similar improvements were also reported in retrospective cohort and comparative studies. Lu et al. (28) reported significantly lower MG-ADL and QMG scores in the treatment group compared with the control group at weeks 4, 12, and 24 (*p* < 0.05), with a higher proportion of patients achieving minimal manifestation status (MMS) at week 24. Lin et al. (22) reported that 75.0% of patients showed clinical improvement at 3 months, and 90.1% maintained improvement at 6 months. Dou (29) reported significantly lower QMG, MG-ADL, and Myasthenia Gravis Composite (MGC) scores in the treatment group compared with the control group at 3 and 6 months (all *p* < 0.05). Zhu et al. (30) reported that the mean QMG score decreased from 15.68 ± 5.42 at baseline to 5.59 ± 3.53 at 3 months, and 20 of 22 patients (91%) achieved minimal symptom expression (MSE) at a mean follow-up of 4 months.

Evidence for telitacicept monotherapy remains limited and was mainly derived from a small retrospective comparative study. In the study by Fang et al. (20), 8 of 10 patients in the telitacicept monotherapy cohort achieved MSE within 4 months, accompanied by reductions in QMG and MG-ADL scores. In comparison, 12.5% of patients in the control group achieved MSE during the same period.

Clinical improvements were also reported in patients receiving sequential or combination treatment strategies. Zhang et al. (21) reported that 6 of 7 patients receiving sequential therapy achieved MR within a mean of 9 weeks. In addition, clinical responses were described across several specific clinical subgroups. Lin et al. (31) observed improvement in 6 of 7 patients with refractory ocular MG, defined as an improvement of at least 2 points on the MG-ADL scale. Mao et al. (32) reported improvement in all pediatric ocular MG cases, while Song et al. (33) observed reductions in QMG, MG-ADL, and 15-item Myasthenia Gravis Quality of Life (MG-QOL-15) scores after 8 weeks of treatment. Clinical improvements were also described in individual cases involving refractory disease, MuSK-Ab-positive MG, and MG associated with autoimmune diseases. However, evidence from these settings remains limited by small sample sizes and the absence of controlled comparisons.

### 3.4 Steroid-sparing effects

Several retrospective studies suggested that telitacicept treatment may be associated with glucocorticoid reduction, although the available evidence was derived primarily from observational studies. Lin et al. (22) reported that the mean daily prednisone dose decreased from 23.53 ± 10.08 mg to 15.63 ± 8.97 mg after 3 months. Zhu et al. (30) reported that the dose had decreased to ≤5 mg/day at the final follow-up, while Dou (29) observed a faster reduction in glucocorticoid dose compared with the control group. In the telitacicept monotherapy cohort of Fang et al. (20), the mean daily prednisone dose decreased from 45.00 mg to 6.25 mg after 4 months. Although these findings suggest a potential steroid-sparing effect of telitacicept, interpretation is limited by the retrospective study designs, small sample sizes, and potential confounding from concomitant therapies.

### 3.5 Safety profile

Overall, telitacicept appeared to be well tolerated in the included studies. Among studies with available safety information, the most commonly reported adverse events included injection-site reactions and upper respiratory tract infections. Most reported adverse events were mild and transient. Serious adverse events were infrequently reported; one randomized phase II study reported a case of pneumonia, while no serious adverse events were reported in the other studies with available safety information. Discontinuation due to adverse events was uncommon, and no treatment-related deaths were reported. However, the interpretation of long-term safety remains limited by the small sample sizes, short follow-up duration, and limited number of controlled studies.

### 3.6 Risk of bias assessment

The overall methodological quality of the included evidence was limited, primarily because most included studies were observational studies, case series, or case reports. According to the Cochrane Risk of Bias tool 2 (RoB 2), the randomized phase II trial was judged as having some concerns regarding risk of bias. Retrospective cohort studies evaluated using the Newcastle–Ottawa Scale (NOS) were assessed as having moderate methodological quality. Case reports and case series evaluated using the Joanna Briggs Institute (JBI) critical appraisal tools were considered to have substantial methodological limitations. The strength of evidence varied across study designs: the randomized phase II trial and comparative studies provided relatively stronger evidence for evaluating treatment effects, while uncontrolled case series and case reports were interpreted more cautiously due to the lack of control groups, small sample sizes, and potential reporting bias. Common methodological limitations included small sample sizes, retrospective study design, lack of control groups, heterogeneity in concomitant treatments, and incomplete outcome reporting. These methodological limitations reduced the overall certainty and generalizability of the available evidence.

## 4 Discussion

### 4.1 Overall interpretation of the evidence

Current evidence suggests that telitacicept may improve disease severity and provide steroid-sparing benefits in patients with MG across different clinical settings. Available studies generally reported improvements in QMG and MG-ADL scores, and a considerable proportion of patients achieved minimal symptom expression (MSE) or minimal manifestation status (MMS). Clinical responses were reported in various MG subtypes, including generalized, ocular, thymoma-associated, pediatric, and refractory MG, indicating the potential therapeutic applicability of telitacicept across heterogeneous patient populations. The strongest support for potential efficacy was provided by the randomized phase II trial and comparative observational studies, whereas findings from uncontrolled case series and case reports should be considered hypothesis-generating due to greater uncertainty. However, these findings should be interpreted cautiously, as the overall evidence remains limited by the predominantly observational nature of available studies and the uncertainty associated with uncontrolled evidence.

The mechanism of telitacicept in the treatment of MG is based on inhibition of the BAFF/APRIL axis, which regulates B-cell survival, differentiation, and pathogenic antibody production (16). This mechanism differs from that of FcRn antagonists, which accelerate IgG clearance, and complement inhibitors, which block the terminal effector pathway at the neuromuscular junction (8, 9). BAFF and APRIL are important regulators of B-cell maturation, survival, and antibody-secreting cell maintenance (39). Through dual inhibition of these pathways, telitacicept may affect multiple stages of pathogenic B-cell responses, including the regulation of autoreactive memory B-cell survival, plasmablast differentiation, and survival signals supporting long-lived plasma cells. This upstream immunomodulatory mechanism may theoretically reduce ongoing autoantibody production rather than only modifying circulating antibody levels. However, the direct effects of telitacicept on specific autoreactive B-cell populations in MG patients remain insufficiently characterized.

The clinical relevance of BAFF/APRIL inhibition may also vary according to MG immunological subtype. Patients with AChR-positive MG, in whom pathogenic antibody production and complement activation play major roles, may theoretically benefit through reduced autoantibody generation (40). In MuSK-positive MG, where pathogenic antibody profiles and underlying immune mechanisms differ from those of AChR-positive MG, the therapeutic effects of upstream B-cell modulation require further investigation. Similarly, the potential role of telitacicept in seronegative MG remains uncertain because the underlying pathogenic mechanisms are heterogeneous and incompletely defined. Supporting this proposed mechanism, several studies included in this review reported decreases in serum immunoglobulin concentrations and CD19^+^ B cell counts during treatment, suggesting a potential effect on B-cell homeostasis (27, 30). These biological changes are consistent with the proposed mechanism of action of telitacicept, although their relationship with long-term clinical outcomes remains to be established. By targeting upstream B-cell dysregulation, telitacicept may offer the potential for broader immunomodulatory effects compared with therapies acting predominantly on downstream immune mechanisms.

### 4.2 Mechanistic rationale and comparison with other targeted therapies

Unlike FcRn antagonists, which can rapidly reduce circulating IgG levels, or complement inhibitors, which block terminal effector pathways at the neuromuscular junction, telitacicept may exert a broader immunomodulatory effect through targeting upstream B-cell biology (8, 9). This distinct mechanism may provide telitacicept with a potential therapeutic role within the current treatment landscape. However, all mechanistic comparisons remain indirect because no head-to-head comparative studies are currently available, and whether this biological distinction translates into superior clinical outcomes remains uncertain.

### 4.3 Clinical implications

An important finding of this review was the reduction in corticosteroid dose observed across multiple studies. Given the well-recognized long-term toxicity of corticosteroids, particularly osteoporosis, metabolic disorders, and increased risk of infection, this steroid-sparing effect may represent an important clinical benefit (3). Although the current evidence remains preliminary and is mainly derived from retrospective studies, telitacicept may represent a potential therapeutic option for selected patients, including those with refractory MG or inadequate response to or intolerance of conventional immunosuppressive therapies.

Given its distinct mechanism of action, telitacicept may provide an additional therapeutic option within the expanding landscape of targeted MG therapies. However, its optimal position in treatment strategies remains uncertain because direct comparative evidence with other targeted therapies is currently lacking.

### 4.4 Strengths and limitations

The available safety evidence should be interpreted cautiously because of the limited sample sizes, short follow-up duration, and small number of controlled studies. Although no major safety signals have emerged, the current evidence remains insufficient to establish the long-term safety profile of telitacicept. Longer-term follow-up is needed to comprehensively assess the risks of infection, persistent immunoglobulin suppression, and other potential delayed adverse effects.

This review has several strengths. It comprehensively summarizes the current clinical evidence on telitacicept, covering multiple MG subtypes and treatment strategies. The inclusion of real-world data from different clinical settings—particularly studies involving refractory MG, pediatric MG, and thymoma-associated MG—broadens the available evidence beyond the currently published clinical trial data. By incorporating data from randomized and observational studies, as well as case series and case reports, this review captures the breadth of current clinical experience with telitacicept in MG.

This review has several limitations, primarily reflecting the limited quality and quantity of the currently available evidence. Most included studies were observational studies, case series, or case reports, with many being retrospective, single-center investigations involving small sample sizes. These study designs are inherently susceptible to selection bias, indication-related confounding factors, and incomplete outcome reporting, thereby limiting the overall certainty of the evidence. Although improvements were consistently reported across studies, the interpretation of these findings should consider the underlying evidence hierarchy. Furthermore, although several studies reported clinically relevant outcomes such as MSE and MMS, the use of heterogeneous outcome measures and inconsistent reporting of responder definitions limited direct assessment of clinical meaningfulness across studies. Results from uncontrolled studies may overestimate treatment effects because of patient selection, regression to the mean, and publication bias. Due to substantial heterogeneity in patient populations, treatment strategies, outcome measures, follow-up duration, and concomitant therapies, a quantitative meta-analysis was not appropriate. Potential participant overlap was systematically assessed in the present review; although overlap was considered unlikely for most included studies, it could not be completely excluded because individual participant-level data were unavailable. All included studies were conducted in China, which may limit the generalizability of the findings to other populations. In addition, the use of combination or sequential treatment strategies in some studies limited the ability to determine the independent contribution of telitacicept to observed clinical improvements. Among the available evidence, the randomized phase II trial and comparative studies provided relatively stronger evidence regarding telitacicept-associated effects. In contrast, findings from uncontrolled combination or sequential treatment settings should be interpreted cautiously because the contribution of concomitant therapies cannot be fully separated.

### 4.5 Future directions

Future research should prioritize larger multicenter randomized controlled trials to confirm these preliminary observations. Head-to-head comparisons with other targeted therapies will be important for defining the relative efficacy, safety, and optimal positioning of telitacicept within current treatment strategies.

Biomarker-based studies may help identify patient subgroups most likely to benefit from treatment and facilitate a more personalized therapeutic approach. Future studies should also clarify the optimal timing and sequencing of telitacicept therapy, including its potential role in sequential or combination treatment strategies across different clinical settings. Long-term studies are needed to evaluate the durability of efficacy and better characterize long-term safety, particularly regarding infection risk and persistent immunoglobulin suppression.

## 5 Conclusion

Available evidence indicates a potential therapeutic role for telitacicept in MG, although the magnitude and durability of clinical benefits remain uncertain. However, these findings are based mainly on limited observational evidence and should be interpreted cautiously. Its dual inhibitory mechanism targeting BAFF and APRIL provides a biologically plausible approach to modulating B-cell-mediated autoimmune responses. Further randomized controlled trials are needed to confirm these preliminary observations and determine the optimal positioning of telitacicept within evolving targeted treatment strategies for MG.

## Data Availability

The original contributions presented in the study are included in the article/Supplementary material, further inquiries can be directed to the corresponding author.
