# Supplementary material for: Telitacicept in myasthenia gravis: a systematic review of clinical outcomes, steroid-sparing effects, and safety
Source: Front Neurol. 2026 Aug 25;17:1916224. doi: 10.3389/fneur.2026.1916224 (PMC13549863; doi:10.3389/fneur.2026.1916224)
Supplement: Supplementary file 1 [file Supplementary_file_1.DOCX]

**Supplementary Table S1.** Characteristics of Studies Included in the Systematic Review

| First author (year) | Study design | Study setting | Inclusion criteria | MG subtype | Sample size (n) | Telitacicept regimen | Treatment duration | Background / concomitant therapy | Follow-up duration |
| --- | --- | --- | --- | --- | --- | --- | --- | --- | --- |
| Yin (2024) | Multicenter, randomized, open-label phase 2 clinical trial | Multicenter, China | Adults with AChR/MuSK-positive generalized MG, MGFA II–IIIb, QMG ≥8, receiving SOC | Generalized MG | 29 | Telitacicept 160 or 240 mg SC once weekly for 24 wks in addition to SOC | 24 wks | Prespecified SOC: pyridostigmine alone; pyridostigmine + one IS; or pyridostigmine + GC + one IS (azathioprine, MMF, MTX, cyclosporine, tacrolimus) | 24 wks |
| Lin (2026) | Single-center retrospective case series | Single center, China | Patients with refractory ocular MG (MGFA I) with typical clinical features and ≥1 positive diagnostic test | Ocular MG (refractory) | 7 | Telitacicept 80–160 mg SC once weekly for a median of 6 mo (IQR 5–8) in addition to SOC | Med 6 mo (5–8) | Pyridostigmine in all pts; GC in most pts; IS (tacrolimus, MMF) in a subset; some had prior IVIG or efgartigimod | Med 7 mo (5–8) |
| Lu (2025) | Single-center retrospective cohort study (PSM) | Single center, China | Patients with MG diagnosed per 2020 Chinese guidelines with clinical features and ≥1 positive test (RNS, antibody, or neostigmine) | Mixed MG | 28 (14 vs 14) | Telitacicept 160 mg SC once weekly for 12 wks in addition to SOC | 12 wks | SOC: GC, IVIG, PE, pyridostigmine, IS (tacrolimus, azathioprine, MMF) | 24 wks |
| Zhang (2025) | Single-center retrospective case series | Single center, China | Adults with generalized MG per 2020 Chinese guidelines with QMG ≥8 or MG-ADL ≥5 and complete follow-up | Generalized MG | 7 | Efgartigimod 10 mg/kg IV weekly ×4 (Wks 0–3), then telitacicept 240 mg SC from Wk 4 with extended intervals (Wks 4, 5, 6, 7, 9, 11, 13, 17, 21, 25) | 21 wks | GC (30–60 mg/d initiated at baseline) and pyridostigmine; no prior long-term IS | 28 wks |
| Lin (2024) | Single-center retrospective cohort study | Single center, China | Patients with refractory generalized MG (MGFA II–V) with inadequate response to ≥2 immunosuppressants or IVIG/PE dependence | Generalized MG (refractory) | 16 | Telitacicept 160 mg SC weekly or biweekly (individualized) as add-on therapy for ≥3 mo | ≥3 mo | GC, tacrolimus, MMF, IVIG/PE, and other IS continued during telitacicept treatment | 3–6 mo |
| Mao (2025) | Single-center retrospective case series | Single center, China | Pediatric patients with ocular MG with typical symptoms and ≥1 positive diagnostic test, excluding other neuromuscular diseases | Ocular MG (pediatric) | 4 | Telitacicept 80 mg SC weekly for 4–8 wks, then biweekly for 8 wks, then monthly maintenance | 4–12 mo | Pyridostigmine, GC, IS (azathioprine, MMF) before or during treatment; one pt also used TCM | 4–12 mo |
| Yang (2025) | Case reports | Single center, China | Patients with MG coexisting with CTD (SLE or Sjögren's syndrome) receiving telitacicept | MG (with CTD) | 3 | Telitacicept 160 mg SC once weekly | Ongoing (≥6–12 mo) | Pyridostigmine, GC, and CTD-related therapies (hydroxychloroquine, leflunomide); some had IVIG, tacrolimus, or other IS | Up to 12 mo |
| Song (2025) | Case series | Single center, China | Patients with refractory generalized MG treated with telitacicept monotherapy | Generalized MG (refractory) | 4 | Telitacicept 160 mg SC once weekly for 8 wks (monotherapy) | 8 wks | Prior pyridostigmine, GC, and IS (tacrolimus, MMF); some had thymectomy or IVIG/PE before treatment | 12 wks |
| Zhang (2023) | Case reports | Single center, China | Patients with refractory generalized MG treated with telitacicept | Generalized MG (refractory) | 2 | Telitacicept 160 mg SC once weekly for 8 wks | 8 wks | Pyridostigmine, GC, tacrolimus, and/or IVIG before or during treatment | 16 wks |
| Qiu (2025) | Case report | Single center, China | Patient with MG complicated by rheumatoid arthritis treated with telitacicept | Generalized MG (with RA) | 1 | Telitacicept 160 mg SC once weekly | NR | MTX and hydroxychloroquine continued; prior biologics: abatacept, adalimumab | ~2 mo |
| Wang (2024) | Case report | Single center, China | Patient with refractory MuSK-positive generalized MG treated with telitacicept | Generalized MG (MuSK-positive, refractory) | 1 | Telitacicept 160 mg SC once weekly for 8 wks; reinitiated in combination with rituximab after relapse | 8 wks (initial) + ongoing (combo) | Pyridostigmine, GC, tacrolimus, MMF, IVIG, PE, and rituximab before or during treatment | Up to 15 mo |
| Zhang (2024) | Case report | Single center, China | Patient with refractory generalized MG receiving telitacicept combined with efgartigimod | Generalized MG (refractory) | 1 | Telitacicept 160 mg SC weekly; efgartigimod 10 mg/kg IV weekly added from Week 28 | ~31 wks | Prednisone, pyridostigmine; prior IVIG, PE, and multiple IS (tacrolimus, rituximab, AZA, MMF, CsA) | ~30 wks |
| Dou (2025) | Single-center retrospective cohort study | Single center, China | Adults with generalized MG (AChR/MuSK positive), MGFA I–IIIb, QMG ≥8, receiving SOC | Generalized and ocular MG | 39 | Telitacicept 160 mg SC once weekly for ≥6 mo in addition to SOC | ≥6 mo | SOC: pyridostigmine, GC, tacrolimus | 6 mo |
| Zhu (2026) | Single-center retrospective cohort study | Single center, China | Patients with AChR-positive generalized MG (including thymoma-associated MG) receiving telitacicept | Generalized MG | 22 | Telitacicept 160 mg SC once weekly for ≥6 mo | ≥6 mo | Prednisone, pyridostigmine, tacrolimus, MMF, AZA, IVIG/PE | 6–12 mo |
| Fang (2025) – telitacicept | Single-center retrospective cohort study | Single center, China | Patients with MG diagnosed per 2020 Chinese guidelines with clinical features and ≥1 positive diagnostic test | Generalized and ocular MG | 10 | Telitacicept 160 or 240 mg SC weekly for 4 wks, then intervals extended | NR | Pyridostigmine, GC, TAC, MMF, AZA | 5 mo |
| Fang (2025) – sequential therapy | Single-center retrospective cohort study | Single center, China | Same cohort as above (sequential therapy subgroup) | Generalized MG | 6 | Efgartigimod 10 mg/kg ×4 (Wks 0–3); telitacicept 240 mg SC from Wk 4 with extended intervals | NR | Pyridostigmine, GC | 21 wks |

*MG, myasthenia gravis; MGFA, Myasthenia Gravis Foundation of America; AChR, acetylcholine receptor; MuSK, muscle-specific kinase; QMG, Quantitative Myasthenia Gravis; MG-ADL, Myasthenia Gravis Activities of Daily Living; SOC, standard of care; SC, subcutaneous; IV, intravenous; PSM, propensity score matching; RNS, repetitive nerve stimulation; CTD, connective tissue disease; SLE, systemic lupus erythematosus; RA, rheumatoid arthritis; wks, weeks; mo, months; Med, median; IQR, interquartile range; GC, glucocorticoids; IS, immunosuppressants; MMF, mycophenolate mofetil; MTX, methotrexate; TAC, tacrolimus; AZA, azathioprine; CsA, cyclosporine; IVIG, intravenous immunoglobulin; PE, plasma exchange; RTX, rituximab; TCM, traditional Chinese medicine; NR, not reported; pts, patients; f/u, follow-up.

†For studies with two arms, values are presented as telitacicept group vs control group. In the "Background / concomitant therapy" column, agents listed are those reported in the original study as permitted or administered during the treatment period.

**Supplementary Table S2.** Baseline Characteristics of Patients Reported in Included Studies

| Study (first author, year) | Age, median (IQR) or mean ± SD, y | Sex | Disease duration, median (IQR) or mean ± SD, mo | MG subtype | Antibody status | MGFA class | Baseline disease severity | Concomitant MG medications |
| --- | --- | --- | --- | --- | --- | --- | --- | --- |
| Yin (2024) | 44.1 ± 12.1 | 13 males, 16 females | 62.2 ± 73.3 | Generalized MG | AChR-Ab positive: 29/29 | II: 16; III: 13 | QMG 18.9 ± 5.2; MG score 24.0 ± 8.9 | Pyridostigmine (29/29); GC (~50%); IS (~40%: TAC, AZA, MMF, MTX, CsA) |
| Lin (2026) | 21 (11–25) | 2 males, 5 females | 98 (46–121) | Ocular MG (refractory) | AChR-Ab positive: 4/7; seronegative: 3/7 | I | QMG 6.0 ± 0.0; MG-ADL 5.7 ± 0.5; MGII-PRO 14.3 ± 2.0 | Pyridostigmine (7/7); GC (most); TAC (4/7); MMF (2/7); prior IVIG (some); prior efgartigimod (some) |
| Lu (2025) | 51.4 ± 14.1 vs 46.9 ± 19.7 | 8 males, 6 females vs 8 males, 6 females | NR | Mixed MG | Seronegative: 2 vs 0; AChR-Ab positive: 10 vs 11; AChR-Ab + Titin-Ab: 2 vs 3 | I: 2 vs 3; II: 6 vs 7; III: 3 vs 3; IV: 3 vs 1 | QMG 6.0 (4.0–10.3) vs 7.0 (4.0–13.3); MG-ADL 4.5 (2.8–6.8) vs 5.5 (3.0–8.3) | GC (14/14); prednisone; TAC; AZA; MMF; IVIG; PE |
| Zhang (2025) | 62 (46–77) | 2 males, 5 females | 7 (1–108) | Generalized MG | AChR-Ab positive: 4/7; MuSK-Ab positive: 2/7; seronegative: 1/7 | IIIa: 3; IIIb: 2; IVa: 1; V: 1 | QMG 12.4 ± 4.3; MG-ADL 7.1 ± 3.6 | Pyridostigmine (7/7); GC (7/7, initial 30–60 mg/d) |
| Lin (2024) | 50.0 ± 17.3 | 8 males, 8 females | 27.6 ± 26.0 | Generalized MG (refractory) | AChR-Ab positive: 14/16; Titin-Ab positive: 8/16; MuSK-Ab positive: 1/16 | IIa: 5; IIb: 5; IIIa: 1; IIIb: 2; IVa: 1; IVb: 1; V: 1 | QMG 14.4 ± 6.9; MG-ADL 5.5 ± 2.9 | GC (15/16); TAC (11/16); MMF (6/16); IVIG (3/16); PE (2/16); RTX (2/16); IL-6R antagonist; MTX; CTX; AZA |
| Mao (2025) | 10.4–13.8 | 2 males, 2 females | 98.4 (32.4–146.4) | Ocular MG (pediatric) | AChR-Ab positive: 2/4; seronegative: 2/4 | I | QMG 0–4; MG-ADL 0–2 | Pyridostigmine (4/4); GC (4/4); AZA; MMF; TCM (1 pt) |
| Yang (2025) | 28, 29, 58 | 1 male, 2 females | 4–24 | MG (with CTD) | AChR-Ab positive: 3/3; Titin-Ab positive: 2/3 | IIIb (Case 1); NR (Cases 2,3) | QMG 13–23; MG-ADL 10–19 | Pyridostigmine; GC; HCQ; LEF; prior IVIG; prior TAC |
| Song (2025) | 59, 61, 62, 69 | 1 male, 3 females | 32–60 | Generalized MG (refractory) | AChR-Ab positive: 4/4 | NR | QMG 9–19; MG-ADL 7–15; MG-QOL15 10–20 | Pyridostigmine; GC; TAC; MMF; prior thymectomy; prior IVIG/PE |
| Zhang (2023) | 64, 70 | 1 male, 1 female | 96; 8 | Generalized MG (refractory) | AChR-Ab positive: 2/2 | IIIb: 1; IVb: 1 | QMG 9–17; MG-ADL 6–12 | Pyridostigmine; prednisone; TAC; IVIG |
| Qiu (2025) | 57 | 1 female | NR | Generalized MG (with RA) | Not reported | III | Ocular deviation | MTX; HCQ; prior abatacept; prior adalimumab |
| Wang (2024) | 70 | 1 female | 3 | Generalized MG (MuSK-positive, refractory) | MuSK-Ab positive | IIIb | QMG 18; MG-ADL 10 | Pyridostigmine; GC; TAC; MMF; IVIG; PE; RTX; prior efgartigimod |
| Zhang (2024) | 37 | 1 female | 156 | Generalized MG (refractory) | AChR-Ab positive; RyR-Ab positive | NR | QMG ~18–20; MG-ADL ~12‡ | Pyridostigmine; prednisone; TAC; RTX; AZA; MMF; CsA; prior IVIG/PE |
| Dou (2025) | 55.5 ± 13.2 | 14 males, 25 females | NR | Generalized and ocular MG | AChR-Ab positive: 39/39; Titin-Ab positive: 16/39; RyR-Ab positive: 12/39 | I–IIIb† | QMG 6 (5–10); MG-ADL 5 (4–7); MGC 6 (4–10) | Pyridostigmine (39/39); GC (39/39); TAC (39/39) |
| Zhu (2026) | 53.8 ± 15.1 | 7 males, 15 females | 13.9 ± 19.0 | Generalized MG | AChR-Ab positive: 22/22 | II: 3; III: 12; IV: 7 | QMG 15.7 ± 5.4; MG-ADL 12.0 ± 4.3; MG-QOL15 22.2 ± 7.1 | Pyridostigmine (22/22); prednisone (22/22); TAC (3/22); MMF; AZA; IVIG/PE (9/22) |
| Fang (2025) – telitacicept | 49.9 ± 23.6 | 4 males, 6 females | 37.0 (4.8–182.0) | Generalized and ocular MG | AChR-Ab positive: 2/10; seronegative: 2/10; not tested: 6/10 | I: 4; IIa: 2; IIIa: 1; IIIb: 2; IVa: 1 | MG-ADL 3.5 (3–9) | Pyridostigmine; GC (9/10 ≥30 mg/d); TAC; MMF; AZA |
| Fang (2025) – sequential therapy | 61.7 ± 10.8 (46–78) | 1 male, 5 females | 36.2 ± 42.0 | Generalized MG | AChR-Ab positive: 4/6; Titin-Ab positive: 2/6; MuSK-Ab positive: 1/6; RyR-Ab positive: 1/6; seronegative: 1/6 | IIIa: 3; IIIb: 2; IVa: 1 | QMG 12 (6–15); MG-ADL 6 (3–7) | Pyridostigmine (6/6); GC (6/6, ≥30 mg/d) |

*NR, not reported. Age data are presented as mean ± SD, median (IQR), median (range), or range, as reported in the original studies. Values for individual case reports are listed as reported. y, years. Disease duration data are presented as mean ± SD, median (IQR), median (range), or range, as reported in the original studies. All values were converted to months. Mo, months.

†For Dou (2025), MGFA class distribution was reported only for the total cohort (I: 42.7%, IIa: 17.7%, IIb: 36.5%, IIIa: 2.1%, IIIb: 1.0%), with no significant difference between groups (P>0.05). The range I–IIIb reflects the enrollment criteria.

‡For Zhang (2024), baseline QMG and MG-ADL values were estimated from graphical data (~18–20 and ~12, respectively); exact baseline figures were not reported in the text.

§Values are presented as telitacicept group vs control group where applicable. Antibody categories are not mutually exclusive. MGFA class is reported as distribution or range depending on data availability.

¶MG, myasthenia gravis; MGFA, Myasthenia Gravis Foundation of America; QMG, Quantitative Myasthenia Gravis; MG-ADL, Myasthenia Gravis Activities of Daily Living; MGII-PRO, Myasthenia Gravis Impairment Index–Patient-Reported Outcome; MG-QOL15, 15-item Myasthenia Gravis Quality of Life; MGC, Myasthenia Gravis Composite; AChR-Ab, acetylcholine receptor antibody; MuSK-Ab, muscle-specific kinase antibody; Titin-Ab, titin antibody; RyR-Ab, ryanodine receptor antibody; GC, glucocorticoids; IS, immunosuppressants; TAC, tacrolimus; AZA, azathioprine; MMF, mycophenolate mofetil; MTX, methotrexate; CsA, cyclosporine; CTX, cyclophosphamide; RTX, rituximab; IVIG, intravenous immunoglobulin; PE, plasma exchange; HCQ, hydroxychloroquine; LEF, leflunomide; TCM, traditional Chinese medicine; IL-6R, interleukin-6 receptor; CTD, connective tissue disease; RA, rheumatoid arthritis; pt(s), patient(s); IQR, interquartile range; SD, standard deviation; y, years; mo, months; mg/d, milligrams per day.

**Supplementary Table S3.** Adverse Events Reported in Included Studies

| Study | N | Any AEs, n/N | Serious AEs, n/N | Discontinuation due to AEs, n/N | Most common AEs |
| --- | --- | --- | --- | --- | --- |
| Yin (2024) | 29 | 26/29 | 1/29 | None | Upper respiratory tract infection (7/29); Blood IgM decreased (7/29); Blood IgA decreased (6/29); Diarrhea (5/29); Blood IgG decreased (5/29); Injection site reactions (5/29); Immunoglobulin decreased (3/29); Urinary tract infection (3/29); Dizziness (2/29); Blood glucose increased (2/29); Hemorrhoids (2/29); Natural killer cell count decreased (2/29) |
| Lin (2026) | 7 | Reported | None | None | Local injection site swelling and pain (2/7) |
| Lu (2025) | 14 | 2/14 | Not reported | None | Injection site rash (1/14); Transient chest tightness (1/14) |
| Zhang (2025) | 11 | Reported | None | None | Upper respiratory tract infections (2/11); Injection site reactions (2/11); Urinary tract infection (1/11); Myalgia (1/11) |
| Lin (2024) | 16 | Reported | None | Not reported | Injection-site pain and swelling (3/16); Herpes zoster (1/16) |
| Mao (2025) | 4 | None | None | None | None reported |
| Yang (2025) | 3 | None | None | None | None reported |
| Song (2025) | 4 | Reported | None | Not reported | Injection-site reaction (1/4) |
| Zhang (2023) | 2 | Not reported | Not reported | Not reported | None reported |
| Qiu (2025) | 1 | None | None | None | None reported |
| Wang (2024) | 1 | Reported | Not reported | Not reported | Injection-site reaction; Herpes zoster infection |
| Zhang (2024) | 1 | Reported | None | Not reported | Injection-site swelling |
| Dou (2025) | 39 | 11/39 | Not reported | Not reported | Upper respiratory tract infection (5/39); Injection-site reactions (3/39); Elevated liver enzymes (2/39); Arthralgia (1/39) |
| Zhu (2026) | 22 | 14/22 | None | None | Injection-site reactions (4/22); Upper respiratory tract infection (3/22); Nausea (2/22); Headache (1/22); Dizziness (1/22); Hypertension (1/22); Diarrhea (1/22); Urinary tract infection (1/22) |
| Fang (2025) | 16 | 1/10 (monotherapy) | Not reported | Not reported | Injection-site pain and swelling (1/10); Upper respiratory infection (1/10) |

*AE, adverse event; IgM, immunoglobulin M; IgA, immunoglobulin A; IgG, immunoglobulin G.

†"Any AEs" and "Serious AEs" are presented as n/N (%) or as "Reported" when described without a total number. "None" indicates that the study explicitly stated no events occurred. "Not reported" indicates that the study did not address this category. For case reports (n ≤ 3), individual AEs are listed when available.

**Supplementary Table S4.** Risk of Bias Assessment of Included Studies

| Study (first author, year) | Study design | Tool used | Overall assessment | Main sources of bias |
| --- | --- | --- | --- | --- |
| Yin (2024) | Multicenter randomized open-label phase 2 clinical trial | RoB 2 | Some concerns | Open-label design with lack of blinding; potential performance bias; limited masking of outcome assessment |
| Lin (2026) | Retrospective case series | JBI | High methodological limitations | Small sample size; absence of control group; inherent selection bias in retrospective case series design |
| Lu (2025) | Retrospective cohort study (PSM) | NOS | Moderate | Retrospective design; potential residual confounding despite PSM; lack of randomization and possible imbalance in unmeasured variables |
| Zhang (2025) | Retrospective case series | JBI | High methodological limitations | Limited sample size; no comparator group; potential selection bias related to patient inclusion and treatment indication |
| Lin (2024) | Retrospective cohort study | NOS | Moderate | Retrospective design; potential selection bias; absence of randomization and control for confounding factors |
| Mao (2025) | Retrospective case series | JBI | High methodological limitations | Small sample size; lack of control group; heterogeneity in treatment duration and prior therapies |
| Yang (2025) | Case reports | JBI | High methodological limitations | Small sample size; absence of comparator; limited generalizability due to heterogeneous comorbid conditions |
| Song (2025) | Case series | JBI | High methodological limitations | Small sample size; lack of control group; potential selection bias and limited external validity |
| Zhang (2023) | Case reports | JBI | High methodological limitations | Small sample size; absence of comparator group; limited ability to infer causality from observational findings |
| Qiu (2025) | Case report | JBI | High methodological limitations | Single-case design; lack of comparator; coexisting rheumatoid arthritis confounding clinical interpretation |
| Wang (2024) | Case report | JBI | High methodological limitations | Single-case design; multiple concomitant and sequential therapies; difficulty in attributing treatment effect |
| Zhang (2024) | Case report | JBI | High methodological limitations | Single-case design; combined use of telitacicept and efgartigimod; limited attribution of efficacy to a single agent |
| Dou (2025) | Retrospective cohort study | NOS | Moderate | Retrospective design; potential selection bias; lack of randomization and limited control for confounders |
| Zhu (2026) | Retrospective cohort study | NOS | Moderate | Retrospective design; potential confounding; absence of randomization and heterogeneity in background therapy |
| Fang (2025) | Retrospective cohort study | NOS | Moderate | Retrospective design; heterogeneity between monotherapy and sequential therapy groups; lack of randomization and control for confounding |

*PSM, propensity score matching; RoB 2, Cochrane Risk of Bias tool (version 2); NOS, Newcastle–Ottawa Scale; JBI, Joanna Briggs Institute critical appraisal tools.

†Overall assessment was based on the criteria of each appraisal tool. The randomized trial was assessed using RoB 2; retrospective cohort and comparative studies using NOS; and case reports and case series using the JBI critical appraisal tools.

**Supplementary Table S5.** Search strategies

Searches were conducted from database inception to January 19, 2026. No language restrictions were applied.

| Database | Search strategy |
| --- | --- |
| PubMed | (("Myasthenia Gravis"[Mesh] OR "Myasthenia Gravis"[Title/Abstract] OR MG[Title/Abstract]) AND ("telitacicept"[Supplementary Concept] OR telitacicept[Title/Abstract] OR RC18[Title/Abstract] OR "TACI-Fc"[Title/Abstract] OR "recombinant TACI-Fc"[Title/Abstract])) |
| Embase | ('myasthenia gravis'/exp OR 'myasthenia gravis':ti,ab OR mg:ti,ab) AND (telitacicept:ti,ab OR rc18:ti,ab OR 'taci-fc':ti,ab OR 'recombinant taci-fc':ti,ab) |
| Cochrane Central Register of Controlled Trials (CENTRAL) | (myasthenia gravis OR MG):ti,ab,kw AND (telitacicept OR RC18 OR "TACI-Fc" OR "recombinant TACI-Fc"):ti,ab,kw |
| Web of Science | TS=("myasthenia gravis" OR MG) AND TS=("telitacicept" OR RC18 OR "TACI-Fc" OR "recombinant TACI-Fc") |
| Scopus | TITLE-ABS-KEY("myasthenia gravis" OR MG) AND TITLE-ABS-KEY("telitacicept" OR RC18 OR "TACI-Fc" OR "recombinant TACI-Fc") |
| China National Knowledge Infrastructure (CNKI) | 主题 = “重症肌无力” AND 主题 = “泰它西普” |
| Wanfang Data | 全部 = “重症肌无力” AND 全部 = “泰它西普” |

**Supplementary Table S6**. Assessment of potential participant overlap among included studies

| **Study** | Institution | Recruitment period | Potential overlap risk | Assessment rationale |
| --- | --- | --- | --- | --- |
| Yin (2024) | Multicenter phase II trial (24 centers, including Wenzhou Medical University) | 2020–2022 | Low–Moderate | Wenzhou Medical University participated in the multicenter trial; however, center-specific enrollment information and direct evidence of participant overlap were unavailable. |
| Lin (2026) | Tongji Hospital | 2024–2025 | Low | Different recruitment period and patient characteristics compared with Lin et al. 2024. |
| Lu (2025) | Henan Provincial People's Hospital | 2023–2024 | Low | Different institution and investigator group. |
| Zhang (2025) | Wenzhou Medical University | 2023–2024 | High | Same institution, overlapping recruitment period, and similar sequential efgartigimod–telitacicept treatment cohort as Fang et al. 2025. |
| Lin (2024) | Tongji Hospital | 2023 | Low | Same institution as Lin et al. 2026; however, recruitment periods and study populations differed. |
| Mao (2025) | Zhejiang University | 2024–2025 | Low | Pediatric MG population from a distinct institution. |
| Yang (2025) | China Medical University | 2023–2024 | Moderate | Same institution as Zhu et al. 2026; however, the study population represented MG with connective tissue disease. |
| Song (2025) | Peking University People's Hospital | 2024 | Low | Independent case series from a different institution. |
| Zhang (2023) | Xuzhou Medical University | 2023 | Low | Independent case report. |
| Qiu (2025) | Zhejiang Provincial People's Hospital | 2024 | Low | Independent RA-associated MG case report. |
| Wang (2024) | Shenzhen Traditional Chinese Medicine Hospital | 2023–2024 | Low | Distinct MuSK-MG population from another institution. |
| Zhang (2024) | Guangzhou University of Chinese Medicine | 2023–2024 | Low | Independent refractory MG case report. |
| Dou (2025) | Shandong Provincial Hospital | 2022–2024 | Low | No other included publication from the same institution was identified. |
| Zhu (2026) | China Medical University | 2022–2023 | Moderate | Same institution as Yang et al. 2025; however, Zhu et al. focused on general MG patients, whereas Yang et al. focused on MG complicated with connective tissue disease. |
| Fang (2025) | Wenzhou Medical University | 2020–2024 | High | Same institution and overlapping recruitment period as Zhang et al. 2025, with similar sequential treatment strategy; partial participant overlap cannot be excluded. |

Potential overlap risk was assessed based on institution, recruitment period, investigator group, study population, and treatment characteristics. As individual participant-level data were unavailable, potential overlap could not be confirmed or completely excluded.
